# *PML-RARA* Fusion Transcripts Detectable 8 Months prior to Promyelocytic Blast Crisis in Chronic Myeloid Leukemia

**DOI:** 10.1155/2020/8830595

**Published:** 2020-09-01

**Authors:** Stephanie Wolanin, Robert K. McCall, Mark J. Pettenati, Michael W. Beaty, Giovanni Insuasti-Beltran, Bayard L. Powell, Stacey S. O'Neill

**Affiliations:** ^1^Wake Forest Baptist Medical Center, Department of Pathology, Winston-Salem, NC, USA; ^2^Molecular Pathology Laboratory Network, Maryville, TN, USA; ^3^Wake Forest Baptist Comprehensive Cancer Center, Section on Hematology and Oncology, Winston-Salem, NC, USA

## Abstract

Promyelocytic blast crisis arising from chronic myeloid leukemia (CML) is rare. We present a 40-year-old male who developed promyelocytic blast crisis 17 months after CML diagnosis, confirmed by the presence of the *t*(15;17) and *t*(9;22) translocations in the leukemic cells. Preserved nucleic acids from routine *BCR-ABL1* testing provided a unique opportunity to evaluate clonal progression over time. Retrospective analysis demonstrated *PML-RARA* fusion transcripts were first detectable 8 months prior to blast crisis presentation. A review of 21 cases of promyelocytic blasts crisis published in the literature reveals a male predominance with earlier age at onset as compared to females. Interestingly, TKI therapy during chronic phase did not impact the time interval between diagnosis and promyelocytic blast crisis. Treatment with standard acute promyelocytic leukemia regimens provides more favorable outcomes with complete molecular remission. Although rare, it is important to consider a promyelocytic blast crisis when evaluating for transformation of CML due to its effective treatment with specific therapies.

## 1. Introduction

Chronic myeloid leukemia (CML) is a myeloproliferative neoplasm defined by the presence of a translocation between chromosomes 9 and 22 leading to the *BCR-ABL1* gene fusion product. The aberrant fusion protein leads to abnormal activation of multiple cellular signaling pathways including JAK/STAT, PI3K/AKT, and Ras/MEK that promote cell growth and survival [1, 2]. Without therapy, progression from chronic phase to accelerated and blast phase is typical. Small molecule tyrosine kinase inhibitors (TKIs) that selectively inhibit the *BCR-ABL1* fusion protein have dramatically prolonged survival of CML patients from an average of 3 years to greater than 8 years and decreased the annual rate of progression to blast crisis from 20% to less than 1% per year [1, 3–5]. While fewer patients continue on to develop blast crisis, the prognosis in blast crisis remains poor, with death generally 3-4 months after diagnosis in pre-TKI era and 7–11 months with TKI therapy [4, 6].

Blast crisis in CML derives from the myeloid lineage in 70% of cases, with the majority of the remaining cases having a lymphoid origin [4]. Rare forms of blast crisis have been described including megakaryocytic, erythroid, monoblastic, eosinophilic, basophilic, and biphenotypic [4]. Promyelocytic blast crisis is a rare variant with <25 cases reported in the literature. Herein, we present a case of CML with promyelocytic blast crisis and review of the literature.

## 2. Case Presentation

The patient is a 40-year-old male with a past medical history significant for classic Hodgkin lymphoma treated with 4 cycles of doxorubicin, bleomycin, vinblastine, and dacarbazine (ABVD) and adjuvant radiation therapy. Approximately seven years after achieving a complete remission, the patient was noted to have asymptomatic leukocytosis (white blood count 44 × 10^3^/*μ*L) during routine follow-up (Table 1). A differential count revealed prominent neutrophilia with maturational left shift and basophilia. No circulating blasts were identified. Hemoglobin and platelets were within normal ranges.

A bone marrow biopsy performed at that time revealed a hypercellular marrow (greater than 95%) with a marked myeloid hyperplasia with maturational left shift and increased myeloid to erythroid ratio. There was no increase in blasts or myelofibrosis. Routine cytogenetics demonstrated presence of the *t*(9;22)(q34;q11.2) translocation in all cells; no other chromosomal abnormalities were detected (Table 1). Fluorescent in situ hybridization (FISH) was positive for *BCR-ABL1* fusion events in 96% of interphase nuclei. Quantitative real-time polymerase chain reaction (RT-PCR) performed on the peripheral blood was positive for p210 *BCR-ABL1* fusion transcripts, with an international scale percent ratio (IS%) of 93.869% (Table 2). Together, the findings supported the diagnosis of chronic myeloid leukemia, *BCR-ABL1*-positive, in chronic phase. The patient was initially treated with imatinib 400 mg daily for 5 months and then reduced for low neutrophils to 300 mg daily for seven months. However, a major molecular response defined as IS% ≤0.1% [7] was not achieved, and therapy was changed to dasatinib 100 mg daily approximately a year after diagnosis secondary to increasing *BCR-ABL1* transcripts by RT-PCR testing. His dose of dasatinib was reduced to 60 mg daily for cytopenias just prior to his diagnosis of blast crisis.

Seventeen months after the initial CML diagnosis, routine laboratory evaluation revealed pancytopenia with circulating immature myeloid cells (Table 1). Clinically, the patient was asymptomatic without splenomegaly or evidence of bleeding. Peripheral blood smear review demonstrated 44% immature cells with rare Auer rods, concerning promyelocytic blast crisis. Coagulation studies showed no definitive evidence of disseminated intravascular coagulation.

**Figure 1:**
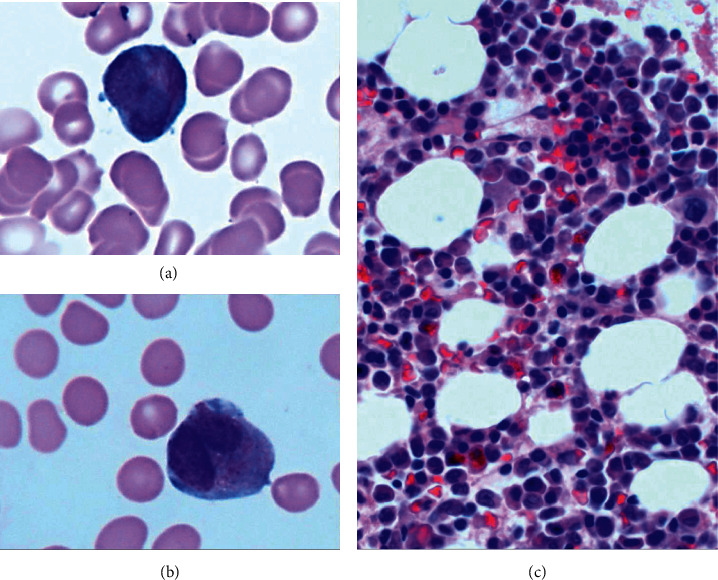
Bone marrow at diagnosis of promyelocytic blast crisis. Promyelocytes with multiple Auer rods (a) and sliding plate nuclear morphology (b) in bone marrow aspirate smears. The bone marrow clot section demonstrates a mildly hypercellular bone marrow with increased promyelocytes (c).

Flow cytometric analysis of peripheral blood demonstrated an immature myeloid population comprising 42% of all cells with expression of CD13, CD33, CD64, CD4, CD117 (dim), CD38, CD45, and cytoplasmic MPO and without expression of CD34, HLA-DR, or CD11c. The bone marrow revealed a mildly hypercellular marrow largely replaced by promyelocytes (67% by manual aspirate differential count; Figure 1). Morphologically, the promyelocytes had a classic appearance with “sliding-plate” nuclei, course cytoplasmic granules, occasional nucleoli, and Auer rods.

**Figure 2:**
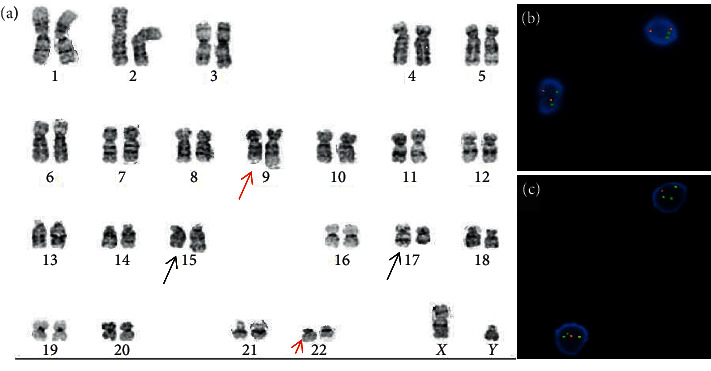
Cytogenetic findings at the time of promyelocytic blast crisis. (a) Conventional karyotype demonstrating *t*(15;17) (black arrows) and *t*(9;22) (red arrows) translocations. (b) FISH probes positive for the *PML-RARA* fusion. (c) FISH probes positive for the *BCR-ABL1* fusion (dual-color, dual-fusion probes).

Routine cytogenetic analysis demonstrated an abnormal cell line containing *t*(9;22)(q34;q11.2) with a simultaneous *t*(15;17)(q22;q21) translocation involving 18 of 20 metaphases examined (Figure 2); the two remaining cells showed a normal male karyotype. FISH was positive for the *BCR-ABL1* and *PML-RARA* fusions in 41% and 47% of interphase nuclei, respectively. Quantitative RT-PCR performed on peripheral blood revealed a p210 *BCR-ABL1* IS% of 49.667% (Table 2). Quantitative RT-PCR for the common breakpoints associated with the *PML-RARA* fusion including bcr1 (long form) and bcr3 (short form) was positive for bcr3 (short form) with a fusion to control ratio of 0.2029 in peripheral blood and 0.2909 in bone marrow; bcr1 was negative in both.

With the aggregate evaluation, a diagnosis of chronic myeloid leukemia in promyelocytic blast phase was rendered. Induction treatment with all-*trans* retinoic acid (ATRA) and arsenic trioxide (ATO) was initiated using the regimen developed by Lo-Coco et al. with arsenic trioxide 0.15 mg/kg intravenously daily and all-*trans* retinoic acid 45 mg per square meter divided into twice daily oral doses [8]; treatment was continued until blood count recovery (42 days). Dasatinib was restarted on day 4 of induction therapy after tolerance, and a stable QTc was confirmed. Dasatinib was held on days 14–16 for pulmonary edema but restarted with aggressive fluid management; it was discontinued prior to discharge on day 42 when repeat imaging showed pulmonary edema, bilateral pleural effusions, and a moderate pericardial effusion. Two weeks after discharge, he was started on nilotinib 300 mg daily, followed in another two weeks by initiation of outpatient consolidation therapy with ATRA 14 days on and 14 days off for seven cycles plus ATO 5 days per week for 4 weeks on and 4 weeks off for four cycles (same doses as induction [8]). The ATRA dose was decreased to 50% beginning with cycle 2 for headaches. Nilotinib was increased to 300 mg twice daily after completion of ATRA and ATO consolidation but was reduced two weeks later after the patient developed rash and dizziness. He is currently on a dose of 150 mg in the morning and 300 mg in the evening. The side effects observed are commonly noted with the individual agents and do not suggest increased toxicities with the combination of a TKI with ATRA and ATO. Fluid retention including pleural and pericardial effusions is well described with dasatinib, and headaches are a common toxicity of ATRA, frequently requiring dose reduction and even discontinuation. His QTc remained stable throughout induction and consolidation therapy.

The patient had a complete morphologic and molecular remission of the promyelocytic blast crisis in less than three months, and RT-PCR for *PML-RARA* transcripts remains undetectable 26 months after diagnosis. With respect to the p210 *BCR-ABL1* transcripts, the patient reached a major molecular response 7 months after diagnosis of blast crisis but has not achieved a complete molecular response to date.

**Figure 3:**
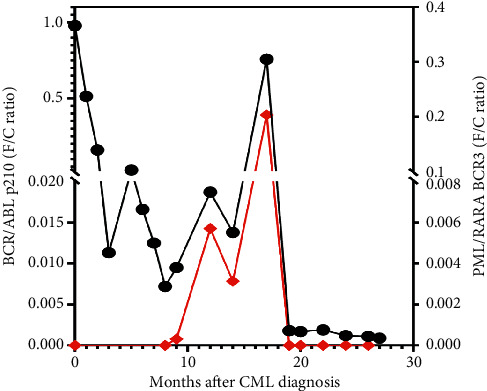
*BCR-ABL1* and *PML-RARA* fusion products over time. CML, chronic myeloid leukemia; F/C, fusion to control ratio.

Since we had been following the patient over time for the p210 *BCR-ABL1* fusion transcript by PCR, we had the unique opportunity to retrospectively evaluate the nucleic acids from peripheral blood samples for the *PML-RARA* fusion product. Analysis of multiple samples demonstrated that the *PML-RARA* bcr3 fusion product was first detectable 8 months prior to the onset of promyelocytic blast crisis (Table 2; Figure 3). The *PML-RARA* transcript level remained at a relatively stable low level for several months leading up to blast crisis diagnosis. Of note, the p210 *BCR-ABL1* transcript trend paralleled the *PML-RARA* transcripts in the progression to blast phase.

## 3. Discussion

Therapies that target the *BCR-ABL1* fusion tyrosine kinase have dramatically improved survival in CML; however, transformation to blast crisis remains a significant cause of mortality. Though fewer patients now develop blast crisis, the prognosis after blast transformation is still poor, with death generally 7–11 months after diagnosis with TKI therapy as compared to 3-4 months in the pre-TKI era [4, 6]. Herein, we describe an unusual variant of blast crisis in which the *PML-RARA* fusion characteristic of acute promyelocytic leukemia (APL) is acquired during progression of the disease and summarize a review of 21 cases of promyelocytic blast crisis of CML in the literature [9–28].

Promyelocytic blast crisis is molecularly analogous to de novo APL, which requires demonstration of the *PML-RARA* fusion by routine cytogenetics, fluorescence in situ hybridization, and/or molecular testing [29]. In our review of the literature, evidence of the *t*(15;17) translocation or *PML-RARA* fusion was provided in 16 of the 21 cases of promyelocytic blast crisis (Table 3). Two additional cases had aberrations in chromosome 17 including an iso(17q) and +17, with a normal chromosome 15 [22, 28]; FISH testing was not performed in either of these cases. Reports of cryptic translocations have been seen in APL cases with iso(17q) [30], raising the possibility that an undetected cryptic translocation may have been present in the case with iso(17q). Additionally, rare APL cryptic translocations have been documented that are negative by karyotype and FISH. In such cases, evidence of the *PML-RARA* fusion is only detectable by PCR and sequence analysis [31]. Other variant translocations including RAR*β* are difficult to detect by routine karyotype [32]. Although uncommon, APL cases without *t*(15;17) may have complex translocations involving chromosomes 15 and 17 and a nontraditional chromosome partner, resulting in a submicroscopic insertion of *RARA* into *PML* [30]. In 3 of the reviewed cases, the promyelocytic blast crisis diagnosis was based on morphologic, immunophenotypic, and/or flow cytometric findings alone; no disease-defining cytogenetic or molecular results were reported [20, 21, 24]. For the purpose of this review, all cases were considered promyelocytic blast crisis of CML, even in the absence of defining reported cytogenetic/molecular evidence.

Review of the 21 promyelocytic blast crisis cases documented in the literature reveals a male predominance, with a male-to-female ratio of 3.2 : 1. This finding is keeping with male predominance in CML [29]. In addition, men developed blast crisis at an earlier average age (38 years ± 17) as compared to women (64 years ± 22 years) (two-tailed *p* value of 0.0127). The interval of time between initial CML diagnosis and promyelocytic blast crisis was less than 1 year in 33% of cases, including two patients who presented with promyelocytic blast crisis at CML diagnosis, 1–5 years in 52% of cases, and greater than 5 years in 14% of cases (average 30 months). This interval of time to promyelocytic blast crisis is similar to the reported time of development of nonpromyelocytic blast crisis (3 years) [4].

Many of the CML promyelocytic blast crisis reports in the literature were published prior to the discovery of TKIs, and accordingly, only 5 of the 19 patients who carried a diagnosis of CML at the time of blast phase were receiving TKI therapy and only one patient was on second-generation TKI therapy. Interestingly, there was no significant difference in time from initial CML diagnosis to promyelocytic blast crisis in patients with and without TKI therapy in this cohort, and the interval ranged from 4 months to 7 years for patients receiving TKI therapy as compared to 10 months to 8 years for patients who were not on TKI therapy (two-tailed *p* value of 0.4964). The data in this cohort suggest that TKI therapy does not prolong the time before progression to promyelocytic blast crisis.

Survival data and/or response to treatment were provided in 16 of the 21 cases. Nine patients died during or within 3 months of the promyelocytic blast crisis diagnosis. Death in these nine patients was primarily due to infection or bleeding events. Survival of patients with promyelocytic blast crisis overall was poor, with only 4 cases resulting in full remission. Such poor outcomes are in contrast to de novo APL in which 90% of patients with the traditional *t*(15;17) are alive and disease-free after 5 years [33]. In our literature review, the treatment regimen was provided for 7 of the 9 patients with survival ≤3 months, and only one patient received APL-specific therapy (e.g., ATRA and ATO). In contrast, 3 of 4 of the patients with complete remission were treated with ATRA; the remaining patient had no documentation of the characteristic *t*(15;17) or *PML-RARA* transcripts [24]. Overall, the data suggest that APL-specific treatment is most successful in achieving remission of promyelocytic blast crisis with documented characteristic cytogenetic/molecular changes.

Herein, we present a unique evaluation of the clonal development of promyelocytic blast crisis in a patient with CML on TKI therapy. At 9 months after initial diagnosis of chronic phase CML, the appearance of low-level *PML-RARA* transcripts was identified by RT-PCR. *PML-RARA* transcripts remained at a stable low level for several months before showing a dramatic increase 8 months after initial detection. In conjunction with this elevation in transcript levels, a marked increase in promyelocytes was appreciated by peripheral blood and bone marrow morphologic evaluation. These findings were consistent with overt promyelocytic blast crisis occurring 17 months after the initial CML diagnosis. With standard APL treatment and continued TKI therapy, the patient has achieved complete molecular remission of the promyelocytic blast crisis. To our knowledge, this is the first report demonstrating the time course of molecular acquisition of *PML-RARA* during chronic phase CML and progression to blast crisis.

Also, of note, is our patient's history of treated classic Hodgkin lymphoma approximately 7 years prior to the diagnosis of CML. This raises the possibility that the CML may be a therapy-related myeloid neoplasm. Although there is an unusual manifestation of a therapy-related myeloid neoplasm, documented cases are present in the literature [34–36]. No additional cytogenetic changes were present at the time of CML chronic phase diagnosis or blast phase to give additional insight into this possibility.

Although therapy-related CML is rare, therapy-related APL (t-APL) is well documented and is most commonly associated with DNA topoisomerase II inhibitor administration [37, 38]. *In vitro* studies have elucidated the mechanism as chemotherapeutic drug-induced DNA topoisomerase II cleavage of double-stranded DNA at the *PML* and *RARA* breakpoint hotspots seen in APL [39]. In our patient, the history of doxorubicin administration for classic Hodgkin lymphoma raises the possibility of a therapy-related *PML-RARA* translocation. In a systemic review, the peak incidence of t-APL following completion of DNA topoisomerase II inhibitor treatment was 2 years, with 95% of cases occurring within 8 years [37]. Our patient presented with promyelocytic blast crisis approximately 8.6 years after completion of ABVD therapy for classic Hodgkin lymphoma. Although this is a long interval, a therapy-related process cannot be excluded. Interestingly, more than one-third of cases in this systematic review were incidentally found on laboratory testing during routine clinical follow-up [37], analogous to our patient. Treatment and outcomes in t-APL are similar to de novo APL [37, 40].

In summary, promyelocytic blast crisis in CML is rare, occurring more commonly in younger males with relatively few cases documented in the literature. Analogous to de novo APL, the *t*(15;17) translocation leads to the aberrant *PML-RARA* fusion protein, and treatment with standard APL regimens provides better outcome in these patients. While the power of this study is limited due to the relatively few published cases available, no previous comprehensive reviews have been published on this subject. Additional studies on these rare cases to further investigate the pathophysiologic mechanisms behind the development of promyelocytic clones, as well as the nidus for the evolution of blast crisis, are warranted. Quantitative RT-PCR has become a routine, highly sensitive tool used not only in the initial diagnosis of APL and CML but also in monitoring patients for treatment response. Since many of these patients are followed by routine *BCR-ABL1* PCR studies for months or years before progression to promyelocytic blast crisis, preserved extracted nucleic acids leading up to the blast crisis are often available, providing a unique opportunity to evaluate clonal development and progression.

## Figures and Tables

**Table 1:** Peripheral blood, bone marrow, and conventional cytogenetic findings over time.

Time relative to CML diagnosis	Peripheral blood				Bone marrow		Conventional cytogenetics
	WBC (×10^3^/*μ*L)	Hemoglobin (g/dL)	Platelets (×10^3^/*μ*L)	Promyelocytes (%)	Cellularity	Promyelocytes (%)	
−7 years	7.2	14.9	200	0	Normocellular	<1	46, *XY* [20]
0	44	14.8	225	0	>95%	1	46, *XY*, *t*(9;22)(q34;*q*11.2) [20]
10 months	3	13.2	106	0	N/A^*∗*^	1	46, *XY*, *t*(9;22)(q34;q11.2) [1]/46, XY [19]
17 months	2	12.8	56	44	70%	67	46, *XY*, *t*(9;22)(q34;q11.2), *t*(15;17)(q22;q21) [18]/46, *XY* [2]

^*∗*^The trephine biopsy was suboptimal and unable to be accessed for cellularity. Reference ranges: WBC 4.8–10.8 × 10^3^/*μ*L, hemoglobin 14.0–18.0 g/dL, platelets 160–360 × 10^3^/*μ*L. CML, chronic myeloid leukemia; WBC, white blood count; N/A, not applicable.

**Table 2:** Quantitative RT-PCR analysis of fusion transcripts in peripheral blood over time.

Months after CML diagnosis	p210 *BCR-ABL1* (IS%)	*PML-RARA* (bcr3 F/C ratio)	WBC (×10^3^/*μ*L)
0	93.869	0	44
1	49.315	ND	3.7
2	15.427	ND	2.6
3	1.085	ND	1.9
5	2.678	ND	2.3
6	1.594	ND	2.6
8	0.691	0	3.7
9	0.567	0.0003	3.2
12	0.879	0.0057	2.3
14	1.174	0.0031	2.7
17^*∗*^	45.328	0.2029	1.7
19	0.107	0	2.5
21	0.138	0	3.8
24	0.070	0	2.9
27	0.074	0	3.5
30	0.104	0	4.1
33	0.045	0	4.2
36	0.081	0	4.8

^*∗*^Blast crisis diagnosis. CML, chronic myeloid leukemia; IS%, international scale percent ratio; F/C, fusion to control; WBC, white blood count; ND, not done.

**Table 3:** Summary of 21 reported cases of promyelocytic blast crisis in CML.

Sex	Age (years)	Treatment during CML chronic phase	Interval between CML diagnosis and blast crisis	Treatment during promyelocytic blast phase	Conventional cytogenetic results	Survival (after blast crisis onset)/outcome	Reference
F	82	Imatinib	2 years	ND	ND^‡^	ND	[9]

M	22	N/A	Concurrent	ATRA, chemotherapy, allogeneic SCT	46, *XY*, *t*(9;22)(q34;q11) [5], 47, *XY*, *t*(9;22), +8, *t*(15;17)(q22;q11-21) [4]^‡^	118 days after allogeneic SCT	[10]

M	31	Cytosine arabinoside, 6-thioguanine^#^	27 months	ND	46, *XY*, *t*(9;22) [40%], 46, *XY*, *t*(9;22), *t*(15;17)(q15;q21) [60%]	3 months	[11]

F	69	Imatinib	13 months	ATRA, idarubicin, Ara-C, imatinib, ATO	46, *XX*, *t*(9;22)(q34;q11.2), *t*(15;17)(q22;q12) [19], 46, *XX* [1]^§‡^	Complete molecular response	[12]

F	85	None^#^	10 months	None	46, *XX*, *t*(9;22), *t*(15;17)(q22;q21) [100%]	2 days	[13]

M	78	Imatinib, dasatinib	7 years	ATRA, ATO	46, *XY*, *t*(9;22)(q34;q11.2), *t*(15;17)(q24;q21) [100%]^§‡^	2 months	[14]

M	32	Imatinib	6 months	ATRA, imatinib	46, *XY*, *t*(9;22)(q34;q11), *t*(15;?;17)(q22;?;q21) [100%]^§‡^	Complete remission	[15]

M	37	Misulban^#^	10 months	Daunorubicin, cytosine arabinoside	46,(9;22), *t*(15;17) [26], 46, *t*(9;22) [5]^*∗*^	ND	[16]

F	52	Hydroxyurea^#^	3 years	Mitoxantrone, etoposide	*t*(9;22)(q34;q11.2), *t*(15;17)(q24;q11.2–12) [9]^*∗*^	6 weeks	[17]

M	55	Hydroxyurea^#^	2 years	ATRA, mitoxantrone, cytosine arabinoside, etoposide	*t*(9;22)(q34;q11.2), *t*(15;17)(q22;q11.2–12) [2]^*∗*^	ND	[17]

M	60	ND^#^	3 years	Cytarabine, mitoxantrone, etoposide, idarubicine, 6-thioguanine	46, *XY*, *t*(9;22)(q34;q11)[40%], 46, *XY*, *t*(9;22)(q34;q11), *t*(15;17)(q22;q21) [60%]^§^	3 weeks	[18]

M	50	ND^#^	3 years	ND	46, *XY*, *t*(9;12;22)(q34;q22;q11) [43.8%], 46, *XY*, *t*(9;12;22)(q34;q22;q11), *t*(15;17)(q22;q12–21) [56.2%]	ND	[19]

M	30	Busulfan^#^	10 months	*N* ^4^-Behenoyl-1-*β*-D-arabinofuranosyl-cytosine, daunorubicin, 6-mercaptopurine, prednisone	46, *XY*, *t*(9;22)(q34;q11) [25%], 46, *XY*, *t*(9;22)(q34;q11), *t*(3;21)(q12;q22) [75%]^◊^	5 months	[20]

F	32	Busulfan^#^	8 years	Doxorubicin	74, *XX*, *t*(9;22), *t*(9;22)(3*n*±)^◊^	1 month	[21]

M	3	Busulfan, alpha-2a interferon, hydroxyurea, cytarabine^#^	3.5 years	“Chemotherapy”	Hypodiploidy, *t*(9;22), iso(17q)^*∗*^^◊^	2 months	[22]

M	38	ND^#^	25 months	Aziridinyl-benzoquinone	46, *XY*, *t*(9;22)(q34;q11) [4], 46, *XY*, *t*(15;17), *t*(9;22) [4], 45, *X*, -*Y*, *t*(15;17), *t*(9;22) [2]	1 month	[23]

M	26	N/A	Concurrent	Cytarabine, arabinoside, daunorubicin, dasatinib, allogeneic SCT	*t*(9;22)^*∗*†^	Complete molecular remission	[24]

M	31	Hydroxy-carbamide, imatinib	4 months	ATRA, ATO, dasatinib, idarubicin, cytarabine, allogeneic SCT	ND^§‡^	Complete molecular remission	[25]

M	27	Natural interferon-*α*	4 years	ND	51, *XY*, +der(1)*t*(1;17)(p11;q11), +7, +8, +8, *t*(9;22)(q34;q11), 22q-^‡^	ND	[26]

M	38	Allopurinol, busulfan	25 months	ND	46, *XY*, *t*(9;22)(q34:q11) [4], 46, *XY*, *t*(15;17)(q22;q12?), *t*(9;22) (q34;q11) [6]	47 days	[27]

M	48	ND^#^	6 years	ATRA	46, *XY* [6]/48, *XY*, +17, +der(1)*t*(1;?)(p13;?), *t*(9;22)(q34;q11) [14]^◊^	>87 days	[28]

^*∗*^Full karyotype information not provided. ^#^Publication date prior to 2001. ^†^FISH studies negative for *PML-RARA* fusion. ^§^FISH studies positive for *PML-RARA* fusion. ^◊^FISH and RT-PCR testing not performed. ^‡^RT-PCR positive for *PML/RARA* fusion. ND, no data provided; N/A, not applicable; ATRA, all-trans retinoic acid; ATO, arsenic trioxide; SCT, stem-cell transplantation.

## Data Availability

The data used to support this study are provided in tables, figures, and references within the manuscript.
